# SUMO1 Promotes Mesangial Cell Proliferation Through Inhibiting Autophagy in a Cell Model of IgA Nephropathy

**DOI:** 10.3389/fmed.2022.834164

**Published:** 2022-03-31

**Authors:** Xia Tan, Yexin Liu, Di Liu, Xiaofang Tang, Ming Xia, Guochun Chen, Liyu He, Xuejing Zhu, Hong Liu

**Affiliations:** Department of Nephrology, Key Lab of Kidney Disease and Blood Purification in Hunan, The Second Xiangya Hospital of Central South University, Renal Research Institute of Central South University, Changsha, China

**Keywords:** SUMO1, autophagy, IgA nephropathy, mesangial cell, proliferation

## Abstract

IgA nephropathy (IgAN) is a common form of primary glomerulonephritis and its main pathological changes are mesangial cell proliferation and matrix expansion. Autophagy inhibition may result in its mesangial cell proliferation and renal lesions. SUMOylation is a eukaryotic-reversible post-translational modification where SUMO is covalently attached to target proteins to regulate their properties. It is largely unclear whether SUMOylation contributes to the pathogenesis of IgAN. This study was designed to investigate the change of protein SUMO1 in mesangial cells of IgAN and its association with autophagy. We found the expression of SUMO1 was upregulated in IgAN, IgA mouse model, and aIgA1-stimulated mesangial cells. In aIgA1-stimulated mesangial cell model, we tested LC3II/I and p62, the autophagy-related proteins suggested the inhibition of autophagy. Inhibited SUMOylation with ginkgolic acid (GA) or silencing SUMO1 could downregulate SUMO1 and SUMO1-p53, promote autophagy, and lessen cell proliferation. In summary, in the mesangial cells stimulated with aIgA1, SUMO1 may contribute to its cell proliferation through inhibited autophagy, and SUMO1-p53 may play a role in this process.

## Introduction

IgA nephropathy (IgAN) is the most common form of primary glomerulonephritis and an important cause of chronic kidney disease and end-stage kidney failure worldwide. A central finding in patients with IgAN is the presence of circulating and glomerular immune complexes comprised of galactose-deficient IgA1. It is characterized by mesangial cell proliferation and mesangial matrix expansion (1). In addition to its well-known multi-hit pathogenesis (2, 3), many factors such as complement activation and autophagy are involved in its occurrence and development (4, 5). Although the pathogenesis of IgAN has been explored at various cellular and molecular levels, no information on protein SUMOylation has been reported in IgAN.

Protein post-translational modifications (PTMs) play essential role in various biological functions. SUMOylation is one of PTMs. SUMOylation modifications are reversible and dynamic processes, in which the modified proteins can be deSUMOylated by SUMO-specific proteases. SUMO includes a family of peptide of 11 kDa, which consists of four informs: SUMO1, SUMO2, SUMO3, and SUMO4. The forms of SUMO2 and SUMO3 are 95% identical in sequence, so they are often grouped together as SUMO2/3 (6). SUMOylation plays crucial roles in chromosomal organization and function, genome stability, proteasomal degradation of proteins and DNA damage repair, and quality control of newly synthesized proteins (7). There is abundant evidence to show that the aberrance of SUMOylation regulation is highly associated with various proliferative diseases that include cancer. But there is no information on protein SUMOylation, which has been reported in IgAN.

Autophagy is a self-degradative process that represents an important physiological catabolic mechanism of the eukaryotic cell. The process of autophagy includes phagophore, autophagosome formation, fusion and degradation, and recycling (8). In the recent years, studies have shown that autophagy plays a crucial role in various cell types, which include neurons, muscles, and cancer cells (9). The molecular control of autophagic activation is dominated by tumor suppressor and oncogene proteins that functionally represent protein kinases (10). The key tumor suppressor protein p53 has numerous tasks, which include regulating cell cycle and autophagy (11). p53 controls the process of autophagy by its own activation or deactivation. By different intracellular positioning, p53 plays diverse roles in autophagy regulation: p53 facilitates autophagy when positioned in the cell nuclei because it can transactivate the two subunits of AMPK as well as TSC2 (12, 13); when positioned in the cytoplasm, p53 inhibits autophagy in three ways: activating autophagy inhibitor mTOR, inhibiting the effect of AMPK, and exerting a direct effect (14, 15). p53 is the target of SUMOylation, and SUMO1 could mediate its transactivation and apoptosis (16). In IgAN, p53 is upregulated in intrinsic renal cells (17). Although the relationships between human disease and autophagy are complicated, there is growing evidence that autophagy is involved in kidney aging and the pathogenesis of kidney disease. In IgAN, the previous study suggested that autophagy inhibition may result in mesangial cell proliferation and renal lesions (5). There are also some studies that showed that the SUMOylation of some proteins is required for autophagosome creation in autophagy and its effects on the regulation of autophagy are complicated (18, 19).

Based on the previous findings, we hypothesized that SUMO1 can promote mesangial cell proliferation through p53's effection of inhibiting autophagy.

## Materials and Methods

### Ethics Statement

The study was approved by the clinical experiment ethical committee of The Second Xiangya Hospital of Central South University, and informed consent was obtained from all study subjects.

### Specimen Collection

Specimens were collected from the patients who were taken renal biopsy in our hospital. A number of 10 patients with IgAN and 10 adjacent patients with minor glomerular abnormality were diagnosed by clinical manifestation and renal biopsy.

### Animals and Experimental IgAN Model

A total of 22 Balb/c rats were purchased from the Hunan SJA Laboratory Animal Co. Ltd. (China) and were bred under controlled environmental conditions. All animal experiments were performed in accordance with the protocols of the Institutional Animal Care and Use Committee at Central South University.

After 1 week of adaptation with general meals and diet balance, the rats were randomly distributed into 2 groups: model group and blank group. The method inducted a successful rat model of IgAN based on our previous studies (5). In brief, rats were induced with continuous oral immunization containing bovine serum albumin (BSA) (Sigma) 800 mg/kg, HCl 8.4 mmol/l in tap water for 18 weeks, followed by subcutaneous injection of castor oil and carbon tetrachloride (CCl_4_) 0.1 ml (5:1) once per week and intraperitoneal injection 0.06 ml once every 2 weeks. In addition, lipopolysaccharide (LPS, 50 μg) was injected *via* tail vein at the 6th and 8th week. For the control group, saline replaced the solvent. The IgAN model was established at the end of the 18th week.

### Immunohistochemical Staining

All the renal staining was performed on 4-μm paraffin sections as we described previously (5). Immunohistochemical stains were performed on formalin-fixed, paraffin-embedded 4-μm sections. Sections were rehydrated and antigens retrieved using heated citrate; primary antibodies were used against the following proteins: SUMO1 (rabbit anti-human, rat, 1:1,000, Cell Signal Technology) or integrin-α8 antibody (mouse anti-human, 1:100, R&D Systems). We used PBS instead of the primary antibodies for negative controls. Then, staining was visualized using horseradish peroxidase (HRP)-coupled secondary antibodies (goat anti-rabbit, 1:500; Abcam). For immunofluorescence, secondary antibodies were coupled to fluorochromes, and nuclei were stained with DAPI (Sigma). The images were acquired using a fluorescence microscope (Nikon Tokyo, Japan) or a confocal laser scanning microscope (Nikon Tokyo, Japan). All immunohistochemical and immunofluorescence analyses were repeated at least 3 times and representative images were presented.

### Cell Culture and Treatment

Human mesangial cells (HMCs) were obtained from the Cell-Bio company and cultured in DMEM supplemented with 10% fetal bovine serum (FBS, Gibco, USA), 100 IU/ml of penicillin, and 100 mg/ml of streptomycin. All cells were maintained at 37°C in a humidified atmosphere with 5% CO_2_. Human mIgA1 was purchased from Abcam company. We incubated the purified mIgA1 at 63°C for 150 min to obtain aggregated IgA1(aIgA1) as described previously (20, 21). The transition from mIgA1 to aIgA1 was monitored using a Sephacryl S-200 column, and a single peak was observed after incubated at 63°C. After cell growth was arrested for 24 h without FBS, HMCs were incubated for 24 h with 25 μg/ml aIgA1 and 24 h with 2 μmol/l ginkgolic acid (GA) or with PBS as control.

### Small Hairpin RNA (shRNA) Plasmids and Cell Transfection

In view of the established characteristics of siRNA-targeting constructs, we designed a pair of siRNA oligonucleotides for SUMO1:5′-GTGACAACACATCTCAAGAATTCAAGAGATTCTTGAGAT GTGTTGTCATTTTTT-3′. The cells were transfected by Lipofectamine 3000 liposome transfection reagent kit of Invitrogen company. HMCs were transfected using control shRNA or shRNA against SUMO1 constructed by Vigene Biosciences Company, following the protocols provided by the manufacturer.

### Real-Time PCR

Total RNA was isolated from cells with TRIzol Reagent (Invitrogen, USA). The first-strand cDNA synthesis was performed using a PrimeScript^TM^ RT reagent kit (Takara, Japan). Real-time PCR was performed to determine relative mRNA levels using a SYBR^R^ Premix Ex Taq^TM^ II (Takara, Japan) on the LightCycler^R^ 96 PCR system (Roche, Switzerland). PCR cycling conditions included an initial step at 95°C for 30 s, followed by 40 cycles of 5 s at 95°C, and 30 s at 60°C. The PCR products were assessed by melting curve analysis, and gene expression levels were calculated using the ΔΔC_t_ method after normalization to the GAPDH housekeeping gene. All PCR samples were tested in triplicate.

### Western Blotting

Cells from different groups were collected, lysed in RIPA Lysis Buffer (Beyotime Biotechnology), and centrifuged at 12,000 g for 10 min at 4°C. The supernatants were collected, and the cellular protein concentrations were determined with a BCA protein assay kit. Protein samples were denatured at 95°C for 5 min, separated by SDS-PAGE, and electrophoretically transferred to polyvinylidene difluoride membranes (Millipore). The blots were incubated with primary antibodies against SUMO1 (1:1,000, rabbit anti-human, Cell Signal Technology), SUMO2/3 (1:1,000, rabbit anti-human, Cell Signal Technology), p53 (1:1,000, mouse anti-human, Cell Signal Technology), cyclin D1 (1:200, rabbit anti-human, Abcam), LC3 (1:1,000, rabbit anti-human, Cell Signal Technology), p62 (1:1,000, rabbit anti-human, Proteintech), or β-actin (1:1,000, mouse anti-human, Abcam) overnight at 4°C. Subsequently, the blots were incubated with an HRP-conjugated secondary antibody (goat anti-rabbit IgG H&L and goat anti-mouse IgG H&L) at room temperature for 1 h, and then, enhanced chemiluminescence was used to visualize the bands.

### Immunoprecipitation

Cell pellets were washed three times with cold PBS and lysed in RIPA buffer for 30 min on ice. After centrifugation at 12,000 rpm for 15 min at 4°C, the lysates were collected and precleared by Protein A/G Plus-Agarose (Santa Cruz Biotechnology) and then incubated with an anti-p53 antibody (1:500, mouse anti-human, Cell Signaling Technology) with continual shaking for 1 h. The protein-antibody complexes were collected with 20 μl of protein A/G plus agarose at 4°C with continual shaking overnight. The next day, the immunoprecipitates were washed three times with lysis buffer and analyzed by SDS-PAGE and immunoblotting.

### Flow Cytometer

Cells in different treatment groups were isolated, fixed in 70% cold ethanol, and stored overnight at −20°C. After washing with PBS, propidium iodide (PI) staining solution (50 μg/ml PI and 100 μg/ml RNase A) was added to the cells and incubated for 30 min in the dark at 37°C. Then, cells were analyzed using flow cytometry (Becton Dickinson Biosciences, USA). FlowJo software was used to analyze the results. Three independent experiments were conducted.

### Statistical Analysis

All statistics were analyzed by SPSS 19.0 statistical software and GraphPad Prism 6.0. Continuous variables are expressed as mean ± standard deviation. The cellular experiments were repeated 5–10 times, and the animal experiments were replicated 5–10 times. Comparison between two groups was detected by *t*-test, whereas comparison among multiple groups was detected by one-Way ANOVA and Kruskal–Wallis for non-parametric test. Difference was considered as statistically significant if *p* < 0.05.

## Result

### Comparison of SUMO1 Expression Between IgAN Kidney Tissues and Adjacent Tissues

**Figure 1:**
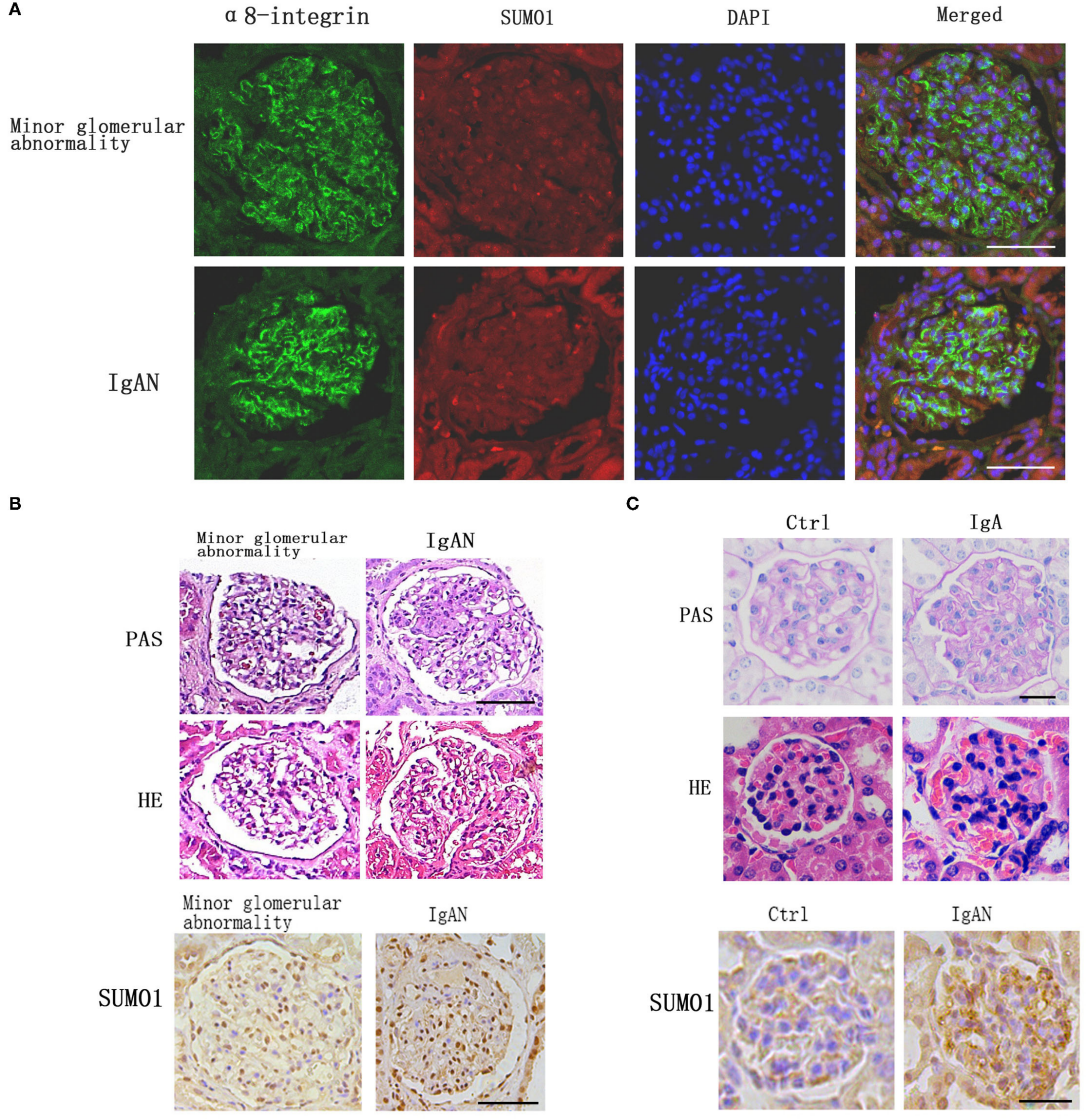
Comparison of SUMO1 expression between IgA nephropathy kidney tissues and adjacent tissues. **(A)** Immunofluorescence staining showed the intraglomerular localization of SUMO1, scale bar 100 μm. **(B)** PAS and HE staining of human kidney tissue. Immunohistochemistry staining for SUMO1 indicated the expression of SUMO1 were up-regulated in IgAN patients group than the minor glomerular abnormality control group, scale bar 100 μm (*n* = 10 per group). **(C)** PAS and HE staining of mouse model kidney tissue. Immunohistochemistry staining for SUMO1 showed the expression of SUMO1 were up-regulated in the mesangial area of IgAN mouse model, scale bar 20 μm (*n* = 11 per group).

Immunofluorescence staining against integrin- α8, a mesangial marker, was used to examine the intraglomerular localization of SUMO1 (Figure 1A). SUMO1-positive areas in the human glomeruli were mostly overlaid with integrin- α8 staining, which suggests the mesangial cellular localization of SUMO1.

Immunohistochemistry staining for SUMO1 indicated that SUMO1 was expressed in human mesangial cells. The expression of SUMO1 was upregulated in IgAN patients' group than the minor glomerular abnormality control group (Figure 1B).

In IgAN mouse model, immunofluorescence demonstrated mesangial IgA deposition. The PAS and HE staining in Figure 1 provide a characteristic overview of the pathologic changes in glomerulus. Compared with control mouses, IgAN mouses were marked with mild mesangial expansion and cellular proliferation in the mesangial area. Immunohistochemistry showed that the expression of SUMO1 was upregulated in the mesangial area of IgAN mouse model (Figure 1C).

### Effects of SUMOylation Inhibitors on HMCs' Autophagy Stimulated With AIgA1

**Figure 2:**
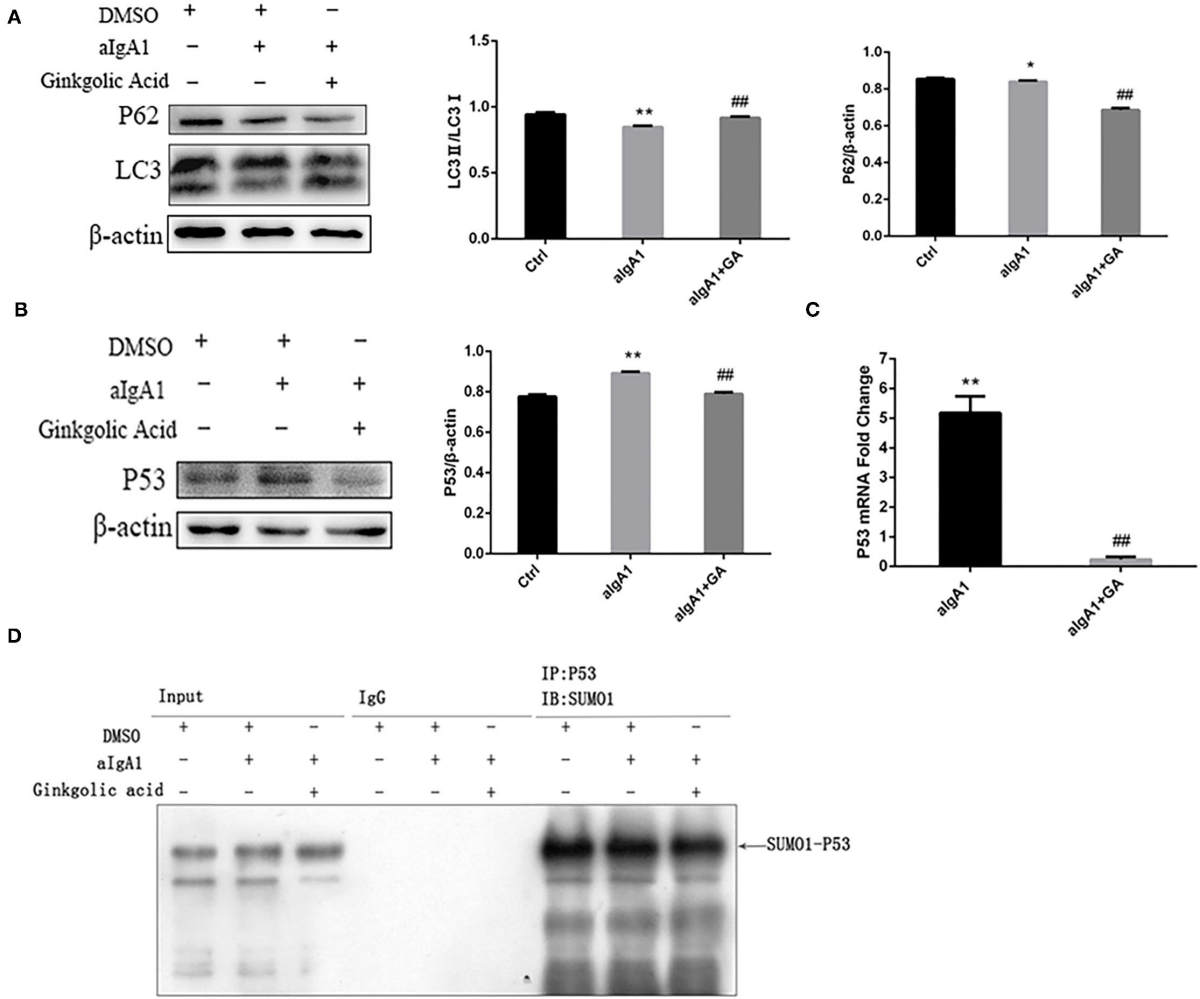
Effects of SUMOylation inhibitors on human mesangial cells' autophagy stimulated by aIgA1. **(A,B)** Western blot analysis for LC3II/I, p62, and p53 in mesangial cells. Cells were treated with aIgA1 and Ginkgolic Acid, and then the whole cells lysates were collected for western bolt analysis. **(C)** Real-time PCR analysis of p53 mRNA. **(D)** Immunoprecipitation for SUMO1-p53 in mesangial cells. Data were presented as mean ± SD (*n* = 10). *p <0.05 vs. control, **p < 0.01 vs. control, ^##^*p* < 0.01 vs. aIgA1 group.

The previous study found that mTOR mediated autophagy inhibition, which result in mesangial cell proliferation in IgAN (5). We investigated the protein SUMOylation and autophagy in cultured HMCs treated with aIgA1 and SUMOylation inhibitor GA. The western blotting (WB) showed that the levels of autophagy-related protein LC3 in mesangial cells were significantly lower in the IgA group (*p* < 0.01) as compared to the levels in the control group; however, it was increased in the IgA+GA group compared to the IgA group (*p* < 0.01). The expression of p62 was upregulated in IgA group than control group (*p* < 0.05), and it was downregulated in the IgA+GA group compared to the IgA group (*p* < 0.01) (Figure 2A). Autophagy-regulated protein p53 was examined. It was increased significantly in the aIgA1 group (*p* < 0.01). Reductions were observed in the expression of p53 (*p* < 0.01) in aIgA1 and GA treatment groups compared to the aIgA1 group (Figure 2B). Real-time PCR showed that the expression of p53 mRNA was upregulated in aIgA1 group (*p* < 0.01). In aIgA1 and GA-treated cells, the expression of p53 mRNA was downregulated (Figure 2C). These results concurred with the results of protein expression. We also tested the expression of SUMO1-p53 by immunoprecipitation. In aIgA1 group, the expression of SUMO1-p53 was upregulated (Figure 2D). Inhibited SUMOylation could downregulate the expression of SUMO1-p53.

### Effects of SUMOylation Inhibitors on HMCs' Proliferation Induced by AIgA1

**Figure 3:**
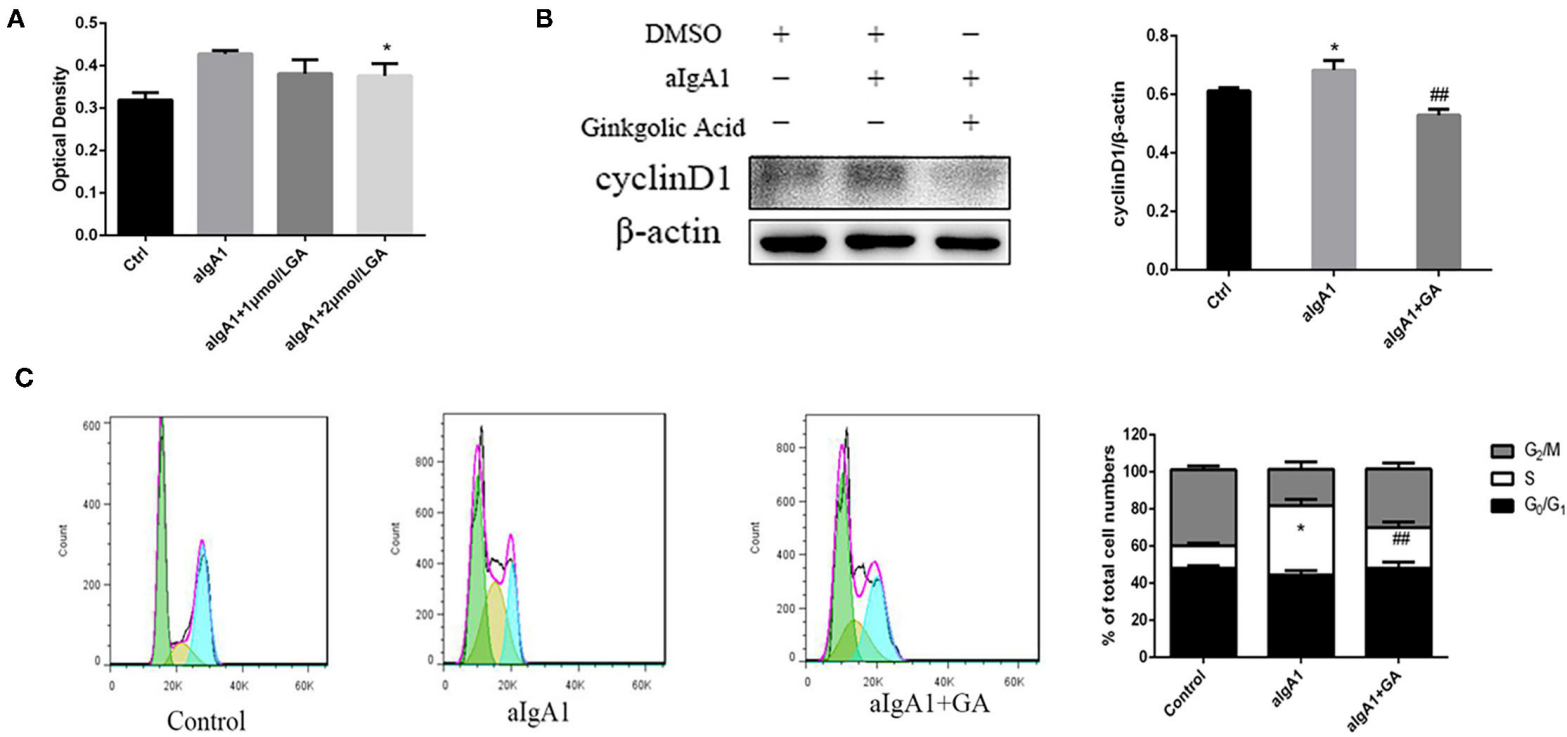
Effects of SUMOylation inhibitors on human mesangial cells' proliferation induced by aIgA1. **(A)** CCK8 analysis of mesangial cells proliferation among groups stimulated with aIgA1 and GA. **(B)** Western blot analysis for Cyclin D1. **(C)** Cell cycle of mesangial cells was analysised by Flow cytometry. Cells were treated with aIgA1 and Ginkgolic Acid. Data were presented as mean ± SD (*n* = 10). *p < 0.05 vs. control, ^##^*p* < 0.01 vs. aIgA1 group.

We examined the proliferation of cells in cultured HMCs stimulated with aIgA1. CCK8 showed that mesangial cell proliferation was observed among groups stimulated with aIgA1. Inhibited SUMOylation with GA could lessen mesangial cell proliferation compared with aIgA1 group (*p* < 0.05) (Figure 3A). The expression of cell cycle protein cyclin D1 (CD1) was examined by WB. Cyclin D1 (*p* < 0.05) was increased significantly in the aIgA1 group. Reductions were observed in the expression of cyclin D1 (*p* < 0.01) in aIgA1 and GA treatment groups compared to the aIgA1 group (Figure 3B). We further examined cell cycle of HMCs treated with aIgA1 and SUMOylation inhibitors by flow cytometry. In aIgA1 group, the S phase of HMC advanced and the period of cell cycle shortened after stimulated with aIgA1. In the aIgA1+GA group, inhibited SUMOylation could recede S phase and extend the period of cell cycle (Figure 3C).

### Blockading SUMO1 in Mesangial Cells Suppresses Cell Autophagy Stimulated With AIgA1

**Figure 4:**
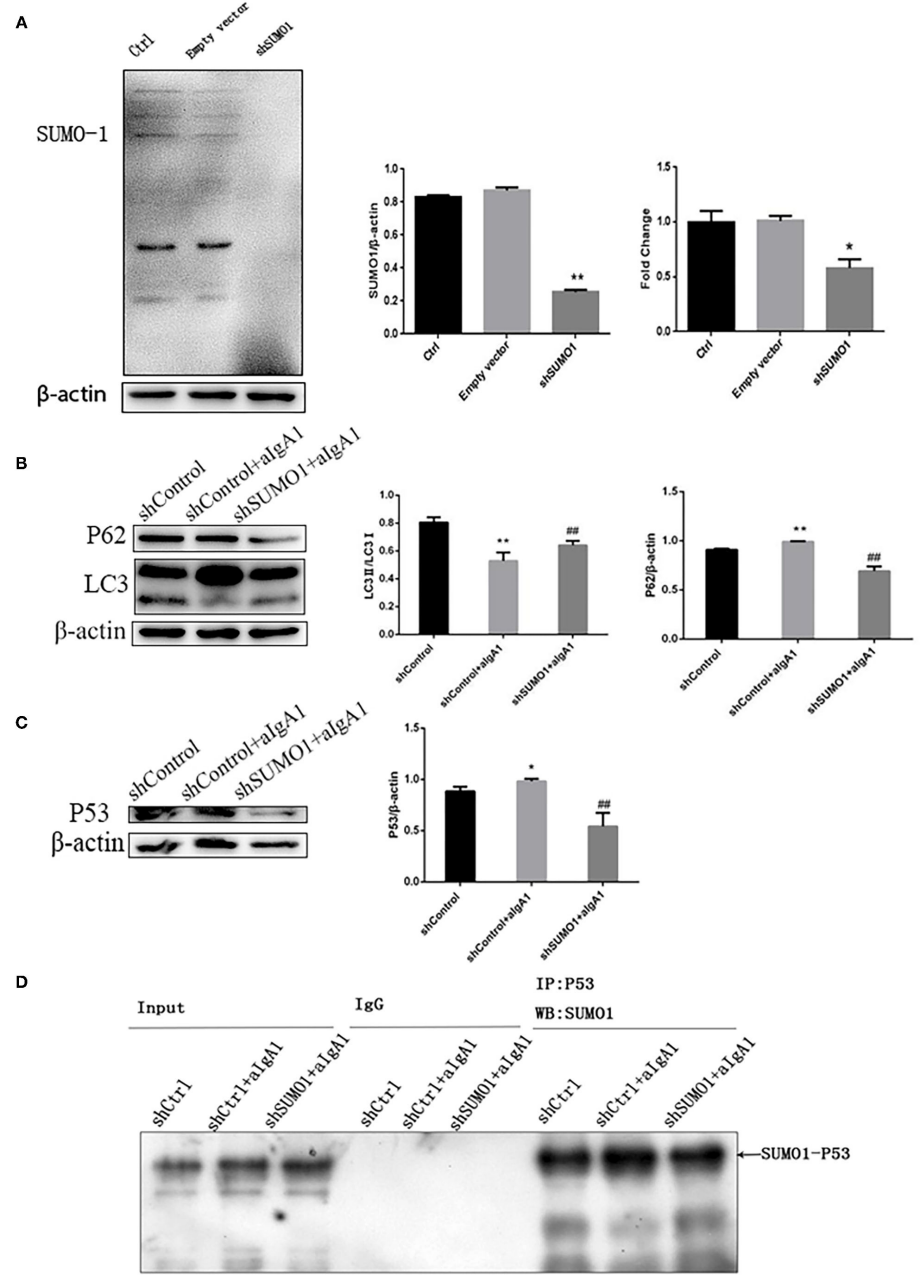
Blockading SUMO1 in mesangial cells suppresses cell autophagy stimulated by aIgA1. **(A)** Western blot analysis and Real-time PCR analysis for SUMO1 in mesangial cells transfected by silencing SUMO1 plasmid. B Western blot analysis for LC3II/I, p62, and p53 in mesangial cells transfected by silencing SUMO1 plasmid. **(C)** Western blot analysis for p53. **(D)** Immunoprecipitation for SUMO1-p53 in mesangial cells. Data were presented as mean ± SD (*n* = 10). *p < 0.05 vs. control, **p < 0.01 vs. control, ^##^*p* < 0.01 vs. shControl+aIgA1 group.

To explore the effect of SUMO1 protein on autophagy and cell cycle of HMC, we further transfected HMCs by silence SUMO1 plasmid than stimulated with aIgA1. Silencing SUMO1 made the expression of SUMO1 proteins (*p* <0.01) and downregulated mRNA (*p* < 0.05) (Figure 4A). The autophagy-related protein LC3 (*p* < 0.01) was upregulated in the silencing SUMO1 with aIgA1-stimulated group, whereas the expression of p62 (*p* < 0.01) was downregulated compared to the aIgA1 group (Figure 4B). WB of mesangial cells revealed that p53 (*p* < 0.01) was significantly downregulated in the shSUMO1+aIgA1 group than in the shControl+aIgA1 group (Figure 4C). We examined the expression of SUMO1-p53 by immunoprecipitation. SUMO1-p53 was downregulated in the shSUMO1+aIgA1 group (Figure 4D).

### Blockading SUMO1 in Mesangial Cells Suppresses Cell Proliferation and Extends Cell Cycle Stimulated With AIgA1

**Figure 5:**
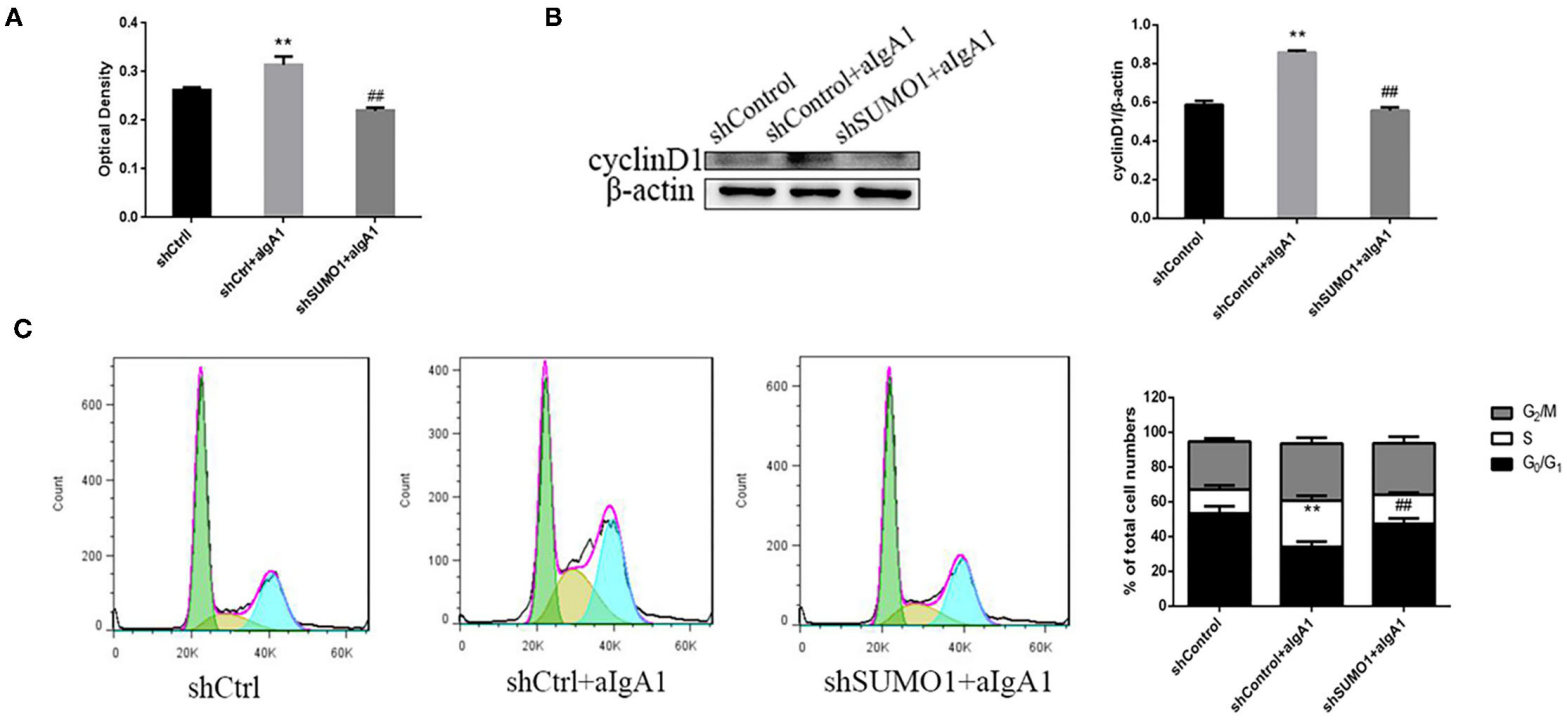
Blockading SUMO1 in mesangial cells suppresses cell proliferation and extends cell cycle stimulated by aIgA1. **(A)** CCK8 analysis of mesangial cells proliferation among groups transfected with control or shSUMO1 plasmids and stimulated by aIgA1. **(B)** Western blot analysis for Cyclin D1. **(C)** Cell cycle of mesangial cells was analysised by Flow cytometry. Cells were transfected with control or shSUMO1 plasmids and stimulated by aIgA1. Data were presented as mean ± SD (*n* = 10). **p < 0.01 vs. shControl, ##*p* < 0.01 vs. shControl+aIgA1 group.

We then examined the proliferation of mesangial cells by CCK8. In the group that cells transfected with control plasmids and stimulated with aIgA1, cell proliferation was promoted significantly (*p* < 0.01). In the group shSUMO1+aIgA1, cell proliferation was lessened than the shControl+aIgA1 group (*p* < 0.01) (Figure 5A). WB revealed that cyclin D1 was downregulated in the shSUMO1+aIgA1 group than in the shControl+aIgA1 group (Figure 5B). The cell cycle was tested by flow cytometry. In the shSUMO1+aIgA1 group, silencing SUMO1 could recede S phase and extend the period of cell cycle compared to shControl+aIgA1 group (Figure 5C).

## Discussion

During the last decade, SUMOylation has been recognized as an important post-translational modification. SUMOylation has been reported to regulate protein subcellular localization, protein-DNA binding, protein–protein interactions, transcriptional regulation, DNA repair, and genome organization. SUMOylation also plays a critical role in autophagy progression. Some targets of SUMOylation implicated in the regulation of autophagy have been identified. One potential target of SUMOylation is the acetyltransferase Tip60/KAT5. Naidu et al. found that SUMOylation of Tip60 may help activate autophagy (22). Another potential target of SUMOylation in regulation of autophagy is PI3KC3/Vps34. SUMOylated Vps34 increases the activity of Vps34 to stimulate autophagy (18, 19). However, given the complexity of autophagy process, the effect of SUMOylation needed more study to identify. Moreover, SUMOylation is known to affect cellular functions in a wild range of disease, which includes cancer, type I diabetes, Crohn's disease, podocyte lesion, and so on (23–26). In kidney disease, SUMOylation determines turnover and localization of nephrin at the plasma membrane (26). Lingyu Wang et al. found that podocytes protect glomerular endothelial cells from hypoxic injury *via* deSUMOylation of HIF-1α (27). In AKI, SUMOylation plays a cytoprotective role (28). In our study, we found in IgAN group and IgA mouse model, the expression of SUMO1 is upregulated in renal cortex. The SUMOylation may trigger mesangial cell proliferation in IgAN. In the study of human mesangial, the expression of SUMO1 was upregulated. Inhibited SUMOylation or SUMO1 could lessen mesangial cell proliferation.

Impaired or deficient autophagy is believed to contribute to proliferative diseases in the previous studies (5). Some factors altered intracellular signaling pathways and autophagy activities under several pathological conditions. In proliferative diseases especially cancer, autophagy modulation is considered to be a double-edged sword. In cancer, autophagy can be neutral, tumor-suppressive, or tumor-promoting in different contexts (29). According to the growing literature, autophagy inhibits malignant transformation (30). Autophagy could buffer metabolic stress in tumor cells (31, 32). Impaired or deficient autophagy is believed to contribute to kidney disease as described in the previous studies that focused on the role of autophagy in kidney disease, especially in acute kidney injury and renal fibrosis (33, 34). In acute kidney injury, autophagy has shown protective properties in ischemic and cisplatin-induced AKI models (33). But in renal fibrosis, the role of autophagy is dual (34). Maybe, that the autophagy can switch roles like in cancer, which depends on the stage of the disease. In IgAN, the previous study showed that autophagy inhibition may result in mesangial cell proliferation and renal lesions (5). In our study, we found that the autophagy-related protein LC3II/LC3 was upregulated, and p62 was downregulated. The aIgA1 could repress the mesangial cell autophagy, shorten the period of cell cycle, and trigger the mesangial cell proliferation.

Inhibited SUMOylation could inhibit SUMO1-p53 and activate autophagy. The key tumor suppressor protein p53 has a number of tasks, which include regulating cell cycle and autophagy (35). P53 controls the process of autophagy by its own activation or deactivation. Recent studies suggest that by different intracellular positioning, p53 plays different roles in autophagy regulation: p53 facilitates autophagy when positioned in the cell nuclei because it can transactivate the two subunits of AMPK as well as TSC2. Meanwhile, p14ARF that exists in the cell nuclei can easily bind with ubiquitin hydrolase MDM2 of p53 to effectively prevent p53 from the degradation caused by MDM2, thus preserving the role of p53 in the nucleus (36); when positioned in the cytoplasm, p53 inhibits autophagy in three ways: activating autophagy inhibitor mTOR, inhibiting the effect of AMPK, and exerting a direct effect (37). As a target of SUMO1, the SUMOylation could mediate p53 transactivation and apoptosis (38, 39). The SUMO1-p53 may play an important role in regulating autophagy. In IgAN, p53 is upregulated in intrinsic renal cells (17). In our study, we found that aIgA1 could repress the mesangial cell autophagy, shorten the period of cell cycle, and trigger the mesangial cell proliferation. Inhibited or silenced SUMOylation could inhibit SUMO1-p53 and activate autophagy. The SUMOylation of p53 in cytoplasm may help to inhibit autophagy.

In conclusion, the expression of SUMO1 and SUMO1-p53 was increased and autophagy was inhibited, and also, the expression of cyclin D1 was increased in aIgA1-stimulated HMC. After GA treatment, SUMO1 and SUMO1-p53 were decreased, and autophagy was increased, while the cell proliferation was lessened. Our findings suggested that overexpression of SUMO1 mediated autophagy inhibition, which may result in mesangial cell proliferation in IgAN, and SUMO1-p53 may play a role in this process.

## Data Availability Statement

The original contributions presented in the study are included in the article/supplementary materials, further inquiries can be directed to the corresponding authors.

## Ethics Statement

The studies involving human participants were reviewed and approved by Medical Ethics Review Committee of The Second Xiangya Hospital of Central South University. The patients/participants provided their written informed consent to participate in this study. The animal study was reviewed and approved by Medical Ethics Review Committee of The Second Xiangya Hospital of Central South University.

## Author Contributions

XTan and HL performed study concept and design. XTan, DL, and YL performed development of methodology and writing. XTang and MX review and revision of the paper. LH provided analysis and statistical analysis. GC and XZ provided technical and material support. All authors read and approved the final paper.

## Funding

This work was supported by research grants (81770714) and (81570622) from the National Natural Science Foundation of China. These funds were used to maintain the database operation, update the statistical software, and purchase reagents and consumables.

## Conflict of Interest

The authors declare that the research was conducted in the absence of any commercial or financial relationships that could be construed as a potential conflict of interest.

## Publisher's Note

All claims expressed in this article are solely those of the authors and do not necessarily represent those of their affiliated organizations, or those of the publisher, the editors and the reviewers. Any product that may be evaluated in this article, or claim that may be made by its manufacturer, is not guaranteed or endorsed by the publisher.
